# Measuring Functional Outcomes After Laser-Based Rehabilitation of Traumatic Scars and Burn Scar Contractures: A Structured Evidence Map and Practical Follow-Up Framework

**DOI:** 10.7759/cureus.117144

**Published:** 2026-09-29

**Authors:** Stefan Bigge, Bianca Bigge

**Affiliations:** 1 Dermatology, Praxis Bigge Ungerechts, Cologne, DEU; 2 Health Sciences, Carl Remigius Medical School, Hochschule Fresenius University of Applied Sciences, Düsseldorf, DEU

**Keywords:** ablative fractional laser, burn scar contracture, outcome measurement, patient-reported outcomes, pulsed dye laser, range of motion, rehabilitation, return to duty, traumatic scar

## Abstract

Traumatic scars and burn scar contractures can cause pain, pruritus, restricted motion, impaired limb function, and difficulty tolerating occupational or protective equipment. Laser-based treatment is increasingly used in rehabilitation, but outcomes remain heterogeneous and often emphasize appearance. This structured narrative review and descriptive evidence map used seven PubMed/MEDLINE search blocks completed on July 21, 2026. Two authors independently screened 898 unique records from 1,191 retrieved records. Reference checking and targeted full-text review supplemented the search; 50 sources informed the framework. Randomized evidence was outcome-dependent: early pulsed dye laser improved patient-rated scar quality in the primary Early Laser for Burn Scars analysis, without consistent observer-rated or quality-of-life benefit. Two within-patient fractional carbon dioxide laser trials reported selected symptom or scar-item improvements despite no significant Vancouver Scar Scale difference. Direct evidence for objective function and return to role remained limited. We propose a minimum-plus-modules framework covering scar characteristics, symptoms, regional function, treatment burden, equipment tolerance, safety and downtime, and graded return-to-work or return-to-duty status. Assessments are linked to baseline, treatment sessions, interim review, treatment completion, and three- and six-to-12-month follow-up. The framework integrates established measures into a practical schedule; it is not a validated core outcome set and requires prospective evaluation.

## Introduction and background

Traumatic scars and burn scar contractures can cause pain, pruritus, dysesthesia, reduced skin glide, tissue fragility, and restricted joint motion. These problems can limit work and social participation even when scar appearance improves. For military personnel and other occupationally active patients, glove or boot wear, load carriage, respirator use, and tool handling may be decisive recovery goals. Military burn care must therefore address durable function as well as survival [1,2].

Laser-based scar treatment, particularly ablative fractional laser (AFL) and vascular-selective pulsed dye laser (PDL), has increasingly been integrated into multidisciplinary scar care. Military dermatology contributed to the transition from cosmetic resurfacing toward rehabilitation-oriented treatment of traumatic scars [3,4]. Consensus statements and reviews subsequently addressed indications, technique, treatment intervals, multimodal care, and safety [5,6]. Systematic reviews describe improvements in scar thickness, pliability, pain, pruritus, and selected functional outcomes, while also emphasizing heterogeneity in scar etiology, device settings, concomitant therapy, assessment methods, and follow-up [7-9].

The remaining problem is how to measure function and participation consistently throughout laser-based rehabilitation. Improvement in color or thickness may coexist with task limitation; conversely, a visibly abnormal scar may be compatible with successful return to work. Scar scales, symptom ratings, functional tests, and participation measures address different constructs and should not be treated as interchangeable.

This review derives a practical follow-up framework for traumatic scars and burn scar contractures in military and civilian occupational settings. It specifies a minimum dataset, optional modules, and treatment-linked measurement times. The framework is not a formal core outcome set, was not developed by Delphi consensus, and does not establish treatment efficacy.

## Review

Search strategy and framework synthesis

This structured narrative review used descriptive evidence mapping to develop an outcome and follow-up framework, rather than to estimate pooled treatment effects. PubMed/MEDLINE was searched through July 21, 2026 using seven predefined thematic blocks (Table 1). No date, language, or publication-type filters were applied. The complete search strings are provided in Appendix A. The original PubMed exports and the PMID-based deduplication record were retained.

**Table 1:** PubMed search blocks and record counts. Counts are recorded as occurrences across overlapping blocks; 898 unique publications remain after PMID deduplication. PMID: PubMed identifier; POSAS: Patient and Observer Scar Assessment Scale; VSS: Vancouver Scar Scale; PROMs: patient-reported outcome measures; ROM: range of motion; RTD: return to duty

Search block	Scope	Records	Screening
1	Main laser, burn/traumatic scar, and outcome search	221	All records
2	Focused laser-plus-burn-scar evidence search	85	All records
3	POSAS, VSS, scar scales, psychometrics, and PROMs	442	All records
4	ROM, goniometry, contracture, and functional motion	167	All records
5	Pain, pruritus, itch, sleep, and symptom outcomes	186	All records
6	Military populations, RTD, and return to work	22	All records
7	Military-journal and novelty scan	68	All records
Total before deduplication	Overlapping search blocks	1,191	All records
Unique after PMID deduplication	892 with abstracts; six without abstracts	898	Independent title/abstract screening by both authors; disagreements resolved by consensus

The searches returned 1,191 records. PMID-based deduplication removed 293 duplicate occurrences, leaving 898 unique records: 892 with abstracts and six assessed from titles and indexing information. Two authors independently screened all titles and abstracts, with disagreements resolved by consensus.

Records were relevant if they informed laser-associated scar outcomes or safety; scar measurement or psychometrics; contracture, range of motion (ROM), or rehabilitation; symptoms or quality of life; or military and occupational participation. Measurement studies were eligible when they informed a prespecified domain, irrespective of whether they evaluated laser treatment. Animal-only studies, acne-scar reports, unrelated laser applications, and acute-care or cosmetic-only reports without relevant outcomes were outside the primary scope. Among relevant sources, anchors were selected for comparative or direct functional evidence, measurement validity or reliability, established outcome frameworks, or distinct clinical information not adequately represented elsewhere. Selection did not depend on a favorable result or statistical significance (Appendix B).

During revision, the archived screening set was reconciled with a record-level anchor-selection audit. Of 898 records, 304 were advanced for further anchor appraisal, and 594 were not advanced. Of the 304, 35 were retained as database-derived anchors; 269 were not selected for citation, mainly because their contribution was represented by another source. Further appraisal used abstracts and available full texts; this stage is not a count of full-text eligibility assessments. The selection flow and reason counts are reported in Figure 1 and Appendix C.

**Figure 1:**
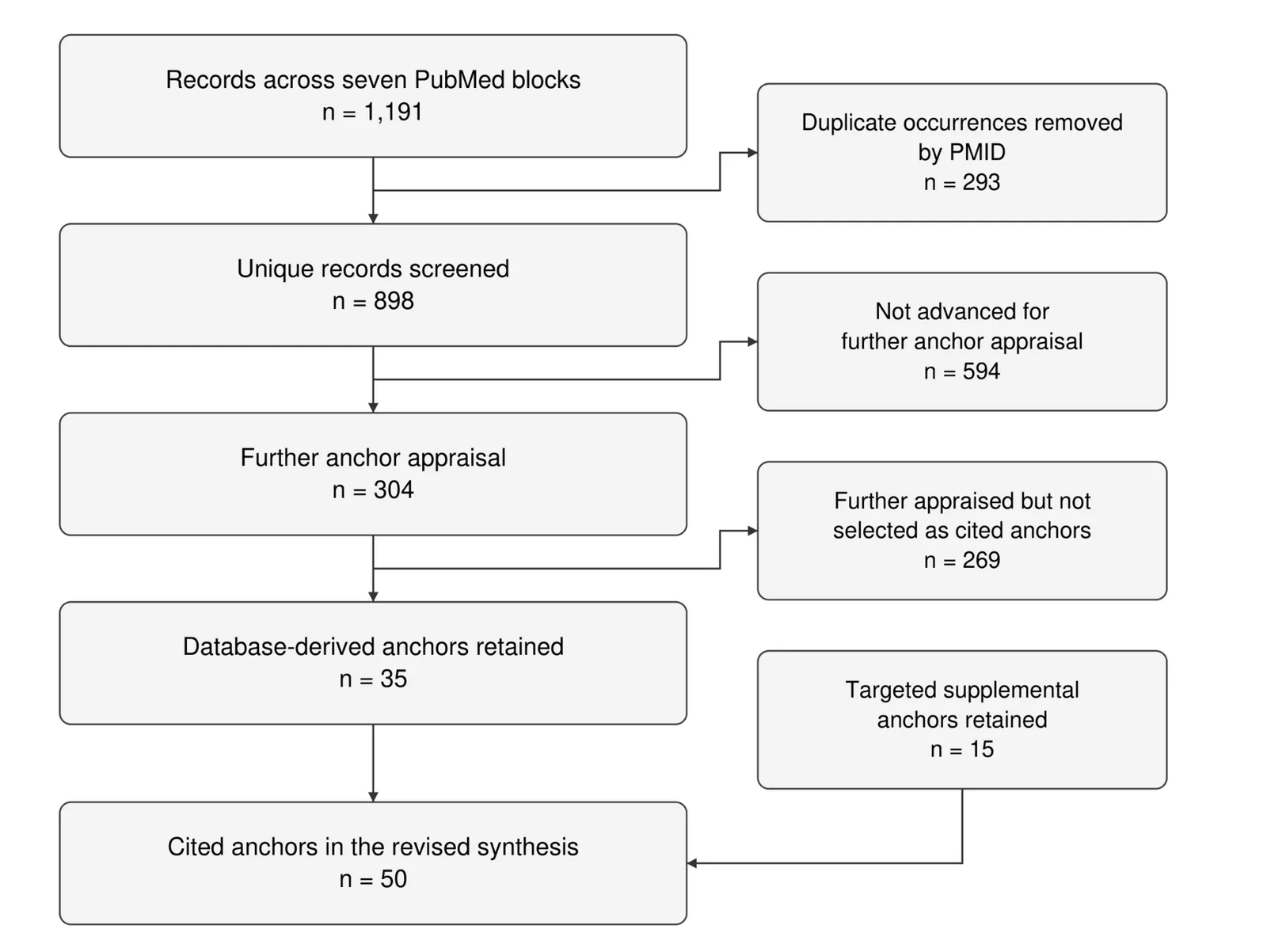
Search and anchor-selection flow. The record-level audit was compiled during revision. Further appraisal used abstracts and available full texts; the 304-record stage is not a full-text count. Appendices C-D provide the reasons and source contributions. The image was created by Stefan Bigge and Bianca Bigge using ReportLab 4.4.9 (ReportLab, Inc., Boston, MA, USA).

Targeted supplementation covered reference lists, the Cochrane Library, original military guidance, and outcome-framework and instrument literature. Fifteen supplemental sources brought the synthesis to 50 cited anchors. Detailed extraction used the full texts of key sources, including the two randomized trials added during revision and three reappraised measurement-methods reports. Appendix D identifies each source and its contribution.

No protocol was registered, no formal study-level risk-of-bias assessment was performed, and no pooled effect was estimated. Certainty judgments from published reviews were retained within their original scope. Primary-study results are reported with the comparison and follow-up specified, adding effect estimates, confidence intervals, and p-values where available; missing paired estimates were not reconstructed from plots.

Framework domains were drawn from the selected clinical, measurement, rehabilitation, and patient-priority literature. Domains were retained for relevance to scar-related disability and feasibility for serial assessment. Equipment tolerance remains exploratory because no validated scar-specific instrument was identified. Table 1 summarizes the search blocks. The revision-stage selection pathway is shown in Figure 1.

Evidence map results

Laser Evidence and Outcome-Specific Effects

The evidence map confirmed a broad and expanding laser-scar literature but a smaller body of work that directly measures objective function or participation. A Cochrane review of 15 randomized trials involving 604 participants concluded that certainty was low or very low for most comparisons, with substantial variation in scar type, laser modality, comparator, and outcome measurement [7]. A systematic review focused on AFL identified 23 studies, 859 patients, and 2,433 treatments; 22 studies reported statistically significant or clinically meaningful improvement, but heterogeneity precluded pooled analysis and durable follow-up was frequently absent [8]. A later function-focused review included 39 studies and 2,602 scars; 36 studies reported improvement in at least one functional or quality-of-life outcome, but most outcomes were subjective and only nine studies reported ROM improvement [9].

The multicenter Early Laser for Burn Scars (ELABS) trial randomized 153 participants and reported 138 complete cases. At six months, early PDL reduced the patient component of the Patient and Observer Scar Assessment Scale (POSAS) by an adjusted mean difference of -4.4 points (95% CI -8.7 to -0.2; P = 0.041). The point estimate was smaller than the six-point difference assumed for sample-size planning. The imputed-data analysis was less conclusive (-4.5 points; 95% CI -9.4 to 0.3; P = 0.068). Observer-rated POSAS, objective color, and quality-of-life outcomes did not show significant between-group differences, and PDL was not cost-effective within the six-month horizon [10].

Two within-patient randomized trials provide complementary evidence for ablative fractional carbon dioxide laser (AFCO2L). Douglas et al. enrolled 20 adults; 19 completed short-term assessment. Six weeks after three treatments, pain (P = 0.047) and itch (P < 0.01) improved in treated areas relative to the pretreatment assessment, while the Vancouver Scar Scale (VSS) did not distinguish treated and control areas. The main report did not provide paired between-area symptom effect estimates or confidence intervals. Only nine patients returned at two to three years, five after additional laser treatment; no long-term POSAS difference was found between areas [11].

In the Early Laser Intervention Promotes Scar Evolution (ELIPSE) trial, 15 adults were enrolled and 14 completed three treatments. No significant difference was found in modified VSS, erythema, pigmentation, or overall patient-POSAS opinion. Patient-rated thickness favored treated scars at three months (P = 0.01) and six months (P = 0.04), and irregularity at three months (P = 0.02). Blinded photographic assessment also favored treated scars (P = 0.002), whereas the Brisbane Burn Scar Impact Profile (BBSIP) improved in both groups. Neither trial established improved joint ROM or return to duty (RTD) [12].

The qualitative study embedded in ELABS described physical, psychosocial, and financial burdens throughout scar management, with participants reporting perceived benefits in adjustment and reintegration [13].

In a longitudinal cohort of 47 adults receiving up to four AFL treatments, scar thickness and all POSAS and BBSIP subscores improved over 12 months. Transient symptoms followed 61 of 89 treatments; infection or delayed healing followed four [14]. In contrast, a Burn Model System comparison of 65 laser-treated and 222 control survivors found no adjusted association with generic outcomes at 12 or 24 months. For Patient-Reported Outcomes Measurement Information System (PROMIS) pain intensity at 12 months, the adjusted laser-group coefficient was 0.30 (95% CI -0.49 to 1.10; P = 0.450) [15]. Differences between studies may reflect measurement sensitivity, case mix, residual confounding, or a lack of meaningful benefit in the outcomes measured.

Direct functional evidence remains less mature than morphologic evidence. In a standardized single-center series of 29 patients evaluated before and after at least three AFL sessions, ROM was the most frequently improved domain, and serial improvements accumulated across treatments [16]. Earlier reports included four patients with function-limiting traumatic scar contractures and a retrospective series of 10 patients with 11 involved joints; the latter reported a mean ROM gain of 19.13 degrees (SD 10.25°; P < 0.02) at six months [17,18]. These studies support the feasibility of objective functional measurement but do not isolate laser effects from natural scar maturation, concurrent rehabilitation, or other interventions. Selected quantitative anchor sources are summarized in Table 2.

**Table 2:** Quantitative evidence map of selected anchor sources. AFL: ablative fractional laser; AFCO2L: ablative fractional carbon dioxide laser; BBSIP: Brisbane Burn Scar Impact Profile; CI: confidence interval; mVSS: modified Vancouver Scar Scale; PDL: pulsed dye laser; POSAS: Patient and Observer Scar Assessment Scale; QoL: quality of life; ROM: range of motion Effect estimates are shown where reported. The main Douglas and ELIPSE reports did not provide paired estimates and confidence intervals for the cited symptom/item contrasts; P-values should not be interpreted as effect sizes.

Evidence source	Quantitative profile	Contribution	Principal limitation
Cochrane review [7]	15 randomized trials; 604 participants	Comparative efficacy and certainty assessment	Low or very low certainty; heterogeneous interventions and outcomes
Buhalog et al. [8]	23 studies; 859 patients; 2,433 treatments	Broad AFL evidence; 22 of 23 studies reported improvement	Marked heterogeneity; limited durable follow-up
Pham et al. [9]	39 studies; 2,602 scars; nine ROM studies	Function-, symptom-, and quality-of-life-focused synthesis	Mostly observational and subjective outcomes
ELABS trial [10]	153 randomized; 138 complete cases; 137 contributed to the six-month patient-POSAS comparison	Six-month patient POSAS: adjusted difference -4.4 (95% CI -8.7 to -0.2); P = 0.041	Imputed analysis CI crossed zero; no significant observer, color, or QoL benefit
Douglas et al. [11]	20 enrolled; 19 short-term; 9 long-term	Treated-area pain P = 0.047; itch P < 0.01 at 6 weeks; no VSS difference	Paired symptom effect estimates/CI absent from main report; 5/9 had further laser before long-term follow-up
Lewis/ELIPSE [12]	15 enrolled; 14 completed; 3-12-month assessments	Thickness: P = 0.01 and 0.04 at 3 and 6 months; irregularity: P = 0.02 at 3 months	No mVSS or overall patient-POSAS difference; BBSIP improved in both groups; small cohort
Betar et al. [14]	47 patients; 89 treatments; 12-month follow-up	Serial POSAS, BBSIP, ultrasound thickness, and safety	Single center; no untreated comparator
Abouzeid et al. [15]	287 survivors; 65 laser-treated	Adjusted 12-month pain coefficient 0.30 (95% CI -0.49 to 1.10); P = 0.450	Observational comparison; residual confounding; no demonstrated generic-outcome association
Travis et al. [16]	29 patients; at least three sessions	Serial objective and subjective assessment; ROM most often improved	No control group; institutional workflow
Shumaker and Xi studies [17,18]	Four-patient case series; 10 patients and 11 joints	Direct task, prosthetic, and ROM outcomes	Small, uncontrolled, concurrent rehabilitation

Prior Serial-Assessment Work

Buhalog et al. recommended baseline, treatment-visit, and final assessment with goniometry, strength, sensibility, imaging, scar scales, and patient-reported outcomes [8]. Travis et al. used repeated POSAS, VSS, durometry, and active ROM measurements during AFL treatment [16]. Our framework extends this established approach to treatment burden, equipment tolerance, and graded return to role; it does not introduce serial assessment itself.

Scar Instruments Measure Different Constructs

Burn core-outcome and rehabilitation frameworks provide an essential conceptual foundation. The international Core Outcome Set for Burn Care Research (COSB-i) initiative defined outcomes important across burn care, while reviews based on the International Classification of Functioning, Disability and Health (ICF) and Delphi work organized recovery across body functions, activities, participation, environmental factors, and personal context [19-21]. These frameworks are broader than laser rehabilitation and should not be replaced. Instead, they support the selection of treatment-linked domains and guard against reducing recovery to scar appearance alone.

Qualitative and consensus work also broadens the outcome space. Interviews with 40 adults and parents identified eight domains important during scar management: scar characteristics and appearance, movement and function, scar sensation, psychological adjustment and sense of normality, body image and confidence, activities, relationships, and treatment burden [22]. A later international Delphi exercise identified 59 scar-related outcomes and emphasized that less frequently measured psychosocial, cost, treatment-understanding, and systemic outcomes may still matter to patients [23]. These findings support a modular framework rather than a single composite score.

Validated scar instruments differ in content. VSS is clinician-rated and emphasizes vascularity, height, pliability, and pigmentation. POSAS adds a patient scale with symptom coverage [24,25]. Confirmatory factor analysis supports reporting physical scar characteristics, pain, and itch separately rather than relying exclusively on a total score [26].

SCAR-Q measures appearance, symptoms, and psychosocial impact across surgical, traumatic, and burn scars [27]. BBSIP covers burn-scar impact on mobility, activities, symptoms, relationships, and treatment and has demonstrated longitudinal validity and responsiveness [28]. These instruments complement rather than replace task performance and participation measures.

Scar assessment has long extended beyond appearance. Brusselaers et al. reviewed 29 publications on clinical scar scales, describing observer dependence, site-relocation problems, and earlier assessment of pain, itch, motion, and activity [29]. Lee et al. reviewed 157 publications on objective methods and recommended combining device-based, subjective, and functional assessment [30]. These are foundations for the proposed framework, not evidence of laser efficacy.

In 34 burn scars from 17 adults assessed by four clinicians, a standardized Cutometer MPA 580 protocol (Courage & Khazaka Electronic GmbH, Cologne, Germany) yielded R0 intraclass correlation coefficients of 0.738 (95% CI 0.609-0.843) for single-rater measurements and 0.918 (95% CI 0.862-0.956) for the average of four raters [31]. R0 represents maximum tissue deformation under suction. The protocol used anatomical landmarks, photographs, and controlled probe pressure. It tested inter-rater reliability within one session, not longitudinal responsiveness. Cutometry can therefore supplement tissue-property assessment but cannot replace symptoms, ROM, task performance, or participation outcomes.

ROM Requires Functional and Anatomical Context

ROM is a logical objective outcome when a scar crosses or influences a joint, but measurement technique matters. Conventional goniometry may underestimate skin-limited motion because standard positioning can slacken adjacent scarred skin. Revised cutaneokinematic positioning detected greater limitation than standard methods in a multicenter burn cohort and can be considered for research or complex contractures when feasible [32]. The term functional ROM should also be used cautiously because the angle needed for one task may be insufficient for another task or for combined planes of motion [33].

Recent work with cutaneous functional units (CFUs) adds an important anatomical dimension. In a prospective multicenter dataset of 307 adults and 7,441 joint motions, the percentage of the relevant CFU burned or grafted, proximal involvement, and age were associated with poorer motion at discharge, whereas total body surface area (TBSA) burned or grafted was not [34]. Prediction models using CFU information outperformed a TBSA-only model in the training dataset, particularly for hand motion, although external validation showed lower discrimination [35]. Baseline characterization should therefore record the involved CFU, its relationship to the joint crease, and the approximate proportion burned or grafted when this information is available.

Contracture prevalence at discharge has ranged from 38% to 54% across studies and generally declines with time, but estimates vary because definitions and measurement methods are inconsistent [36]. ROM should consequently be paired with a region-specific task or instrument. QuickDASH has been evaluated after upper-limb burns, and grip or pinch dynamometry can add objective information when hand strength is relevant [37,38].

Symptoms, Mental Health, and Treatment Burden Modify Interpretation

Pain and pruritus are clinically important outcomes rather than ancillary scar symptoms. A systematic review of 15 randomized trials involving 780 participants found low-to-moderate-certainty evidence that laser, massage, and shockwave interventions can reduce burn-scar pain and pruritus, while effects on elasticity and vascularity were less clear [39]. A zero-to-10 intensity rating is feasible at every visit; the 5-D Itch Scale or a pain-interference instrument can be added when duration, distribution, sleep disruption, or activity interference requires greater detail [40].

Mean symptom changes can conceal heterogeneous responses. A retrospective study classified 56.4% of patients as pain nonresponders and 55.1% as itch nonresponders using a three-point improvement threshold; responders improved mainly after the first treatment and had higher baseline scores [41]. Mental-health diagnosis and gabapentin exposure were associated with symptom trajectories in another cohort [42]. Baseline severity, medication use, mental-health context, and responder proportions should therefore accompany mean scores. Such observational associations should not be treated as causal predictors.

Treatment burden is also an outcome. Travel, procedure time, anesthesia or sedation, wound care, time away from work or duty, family impact, costs, and willingness to continue can determine whether a clinically effective pathway is acceptable. These elements should be recorded briefly after each session and at treatment completion, particularly when repeated procedures are planned.

Safety and Downtime Are Functional Outcomes

Safety assessment should extend beyond infection. A regional center reported more than 1,800 laser treatments in 414 patients, with sedation-related prolonged recovery identified as a workflow concern [43]. Infection or delayed healing occurred after four of 89 treatments in the longitudinal cohort above [14]. A retrospective series reported no association between prophylactic antibiotics and infection prevention (P = 1.0) [44]. This does not establish equivalence between antibiotic strategies; antibiotic exposure and infectious outcomes should be recorded.

Relevant safety and downtime variables include epithelialization time, blistering, dyspigmentation, new ulceration, scar worsening, postoperative pain, nausea, prolonged recovery, unscheduled care, unplanned admission, interruption of therapy, and days unable to use essential equipment or perform required work. These variables should be reported per treatment session as well as per patient because repeated-session risk is otherwise obscured.

Military and Occupational Outcomes Require Graded Definitions

Military RTD is not equivalent to complete functional recovery. In a cohort of 285 combat-related burn survivors, 190 returned to duty, and 95 were medically discharged; burn size, hospitalization, and inhalation injury were more predictive than the mere presence of a hand burn [45]. Among 101 service members with upper-extremity burns, a proposed Burn Scar Contracture Severity Scale changed over time and discriminated RTD from non-RTD at a study-specific cutoff of 6.5, but correlations with DASH and American Medical Association impairment scores were weak, and the cutoff was not externally validated [46].

Other military research confirms that self-reported RTD may coexist with persistent deficits and should not be used as a stand-alone proxy for full function [47]. A 2026 scoping review of military trauma identified 481 studies, 297 distinct patient-reported outcomes, and 314 PROM families, with substantial heterogeneity and relatively little burn-specific work [48]. Civilian return-to-work literature is similarly heterogeneous and influenced by injury severity, treatment duration, psychosocial factors, job demands, and workplace accommodation [49]. Role status should therefore distinguish unrestricted return, restricted return in the same role, modified role, nonreturn, and separation, while separately documenting deployability, sustained performance, and time to return. The relationship between existing doctrine, prior art, and the proposed framework is summarized in Table 3.

**Table 3:** Relationship to existing doctrine, prior art, and outcome frameworks. JTS: Joint Trauma System; CPG: Clinical Practice Guideline; ROM: range of motion; COSB-i: Core Outcome Set for Burns-international; ICF: International Classification of Functioning, Disability and Health; CFU: cutaneous functional unit; PROM: patient-reported outcome measure; RTD: return to duty

Source or framework	What it covers	Residual gap	Role of the proposed framework
JTS Burn Care CPG [2]	Acute and deployed assessment, resuscitation, wound care, teleconsultation, definitive care, and evacuation	No post-healing laser outcome schedule or graded return-to-role pathway	Adds a downstream measurement overlay without replacing acute doctrine
Laser reviews and consensus [3-9]	Indications, modalities, technique, safety, scar and symptom outcomes	No unified minimum dataset spanning function, burden, equipment, and role	Organizes outcomes rather than prescribing laser parameters
Serial laser assessment [8,16]	Baseline, treatment-visit, and final scar, ROM, and patient-reported assessments	Limited occupational and participation content	Extends prior serial measurement to treatment burden, equipment, and graded role status
COSB-i and ICF work [19-21]	Broad burn outcomes, activities, participation, and context	Not linked to individual laser treatment episodes	Translates established domains into a compact visit-linked dataset
Scar scales, measurement studies, and patient-priority work [22-31]	Morphology, symptoms, impact, measurement properties, and earlier motion/activity assessment	No validated treatment-linked dataset spanning tissue properties, specific tasks, and sustained role performance	Standardizes assessment context and combines complementary measures without treating them as equivalent
ROM and CFU research [32-36]	Joint motion, positioning, functional anatomy, contracture prevalence, and prediction	Not consistently integrated with symptoms, safety, or participation	Links motion to task, equipment, and role outcomes
Military PROM and RTD literature [45-49]	Duty disposition, PROM heterogeneity, role change, and return to work	Definitions, timing, and instruments remain inconsistent	Uses graded role status and separates RTD from full recovery

Proposed minimum-plus-modules framework

Design Principles

The minimum dataset is intended for repeated clinical use, with additional modules selected by anatomy, symptoms, occupational demands, and study purpose. No patient needs every instrument. Patient-reported subscales can be prioritized when time is limited, with observer scales and objective testing added when appropriate.

Interpret morphology, symptoms, function, and participation separately. Select regional tasks before treatment, link reassessment to defined treatment episodes, and supplement binary return-to-work or RTD with restrictions and sustained performance. Table 4 presents the dataset; Table 5 gives region-specific modules.

**Table 4:** Proposed minimum-plus-modules outcome dataset. CFU: cutaneous functional unit; POSAS: Patient and Observer Scar Assessment Scale; PROM: patient-reported outcome measure; BBSIP: Brisbane Burn Scar Impact Profile; T0: baseline assessment; T1: treatment-cycle assessment; T2: early follow-up; T3: intermediate follow-up; T4: late follow-up; T5: final follow-up; ROM: range of motion; QuickDASH: Quick Disabilities of the Arm, Shoulder and Hand; RTD: return to duty; T0: pretreatment baseline; T1: each treatment cycle; T2: interim review; T3: treatment completion; T4: approximately three months after completion; T5: six to 12 months after completion

Domain	Field-ready minimum	Optional enhanced module	Timing	Interpretive caution
Scar and treatment context	Etiology; anatomical region, CFU, and defined assessment site; scar age and maturity; proportion of relevant CFU burned or grafted when available; graft status; prior reconstruction; previous treatment; laser modality and session number	Standardized photography, ultrasound, three-dimensional imaging, cutometry with site relocation and probe-pressure control [29-31], detailed dosimetry	T0; update at T1 when status or treatment changes	Context determines which modules are relevant and supports reproducibility
Patient-reported scar burden	POSAS Patient Scale or a concise scar-specific PROM; report physical scar items, pain, and itch separately	POSAS Observer Scale, SCAR-Q, or BBSIP for burn-specific impact [25-28]	T0, T2, T3, T4, T5	A total score should not conceal symptom domains or replace function
Pain, pruritus, and sleep	Zero-to-10 pain and itch ratings; medication use; sleep interference when present	Neuropathic pain or pain-interference tool; 5-D Itch Scale [39-42]	T0 and every T1; repeat T2-T5	Document baseline severity and responder status; mental-health context may affect perception
ROM and regional function	Active joint-specific ROM when relevant plus one predefined regional task	Revised cutaneokinematic goniometry, QuickDASH, grip or pinch strength, dexterity, gait, endurance, therapist observation [32-38]	T0, T2, T3, T4, T5	Standardize position and distinguish skin restriction from tendon, joint, weakness, and pain
Equipment tolerance	Item or interface; wear duration; pain or itch; skin breakdown; fit or seal problem; effect on task	Structured diary, supervised wear test, interface-pressure mapping where available	T0, T2, T3, T4, T5	Exploratory and nonvalidated; not a substitute for formal equipment certification
Role and participation	Preinjury role demands and current status: unrestricted, restricted same role, modified role, not returned, or separated	Deployability, medical-board status, time to return, sustained performance, work accommodations	T0, T3, T4, T5	RTD or return to work is not synonymous with full recovery [45-49]
Safety and downtime	Epithelialization time; infection; blistering; dyspigmentation; breakdown; scar worsening; days unable to work, train, use equipment, or attend therapy	Sedation and anesthesia details, nausea, prolonged recovery, unscheduled care, admission, antibiotic use, standardized adverse-event grading [14,43,44]	Each treatment cycle (T1) and T2-T5	Report events per treatment and per patient; relate downtime to role requirements
Treatment burden	Procedure time, travel, wound-care burden, time away from work or duty, willingness to continue	Cost, family impact, productivity loss, qualitative interview	Each treatment cycle (T1); T3 and T4	Clinical benefit may not translate into an acceptable pathway [13,22,23]
Quality of life and psychosocial context	Brief baseline assessment of current burden; global change at follow-up; relevant mental-health context	SCAR-Q psychosocial scale, BBSIP, or a validated generic instrument when cross-condition comparison is needed	T0, T3, T4, T5	Generic measures complement but should not replace scar- and task-specific outcomes [14,15,22,27,28]

**Table 5:** Region- and mechanism-specific functional modules. QuickDASH: Quick Disabilities of the Arm, Shoulder and Hand; ROM: range of motion; CFU: cutaneous functional unit

Region or mechanism	Core functional question	Useful optional measures	Examples of role-relevant tasks
Hand, wrist, or upper limb	Can the patient grasp, manipulate, lift, and sustain repetitive use?	QuickDASH; grip or pinch dynamometry; dexterity; composite digit ROM; sensibility	Glove wear; tool or weapon handling; patient care; lifting and carrying
Neck or face	Can the patient achieve required motion and tolerate facial equipment?	Cervical ROM; mouth opening; facial movement; symptom-provocation test	Helmet use; visual scanning; respirator or mask fit; communication
Axilla, shoulder, or torso	Can the patient reach, elevate the arm, rotate the trunk, and tolerate external pressure?	Shoulder ROM; task observation; chest-wall symptoms; CFU mapping	Load carriage; body armor; overhead work; transfers
Lower limb or foot	Can the patient walk, climb, squat, kneel, and tolerate footwear under load?	Knee and ankle ROM; gait; sit-to-stand; step or endurance test	Boot wear; marching; stairs; kneeling; prolonged standing
Amputation or prosthetic interface	Can the skin tolerate progressive prosthetic loading without breakdown?	Prosthetic wear time; skin checks; gait; socket-pressure mapping	Prosthetic training; prolonged wear; uneven-ground mobility
Focal non-burn traumatic scar	Does pain, dysesthesia, adherence, fragility, or tissue tethering impair a specific task?	Sensory testing; soft-tissue glide; task-provoked pain; standardized photography	Harness or equipment contact; repeated occupational motion; scar-specific task

Region- and Mechanism-Specific Modules

Select the module that matches the affected region or injury mechanism; not every patient requires every module (Table 5).

Treatment-Linked Follow-Up Schedule

T0 is the pretreatment baseline. T1 covers each treatment cycle: a pre-session check, followed by documentation of healing, adverse events, symptom change, treatment burden, and resumption of rehabilitation. T2 is an interim review after approximately two to three sessions, or earlier if needed for a treatment decision. T3 is treatment completion; T4 at approximately three months and T5 at six to 12 months after completion assess durability, late adverse effects, equipment use, and sustained role performance. These intervals are proposed assessment windows, not validated treatment intervals, and may be adapted to healing and clinical need (Figure 2).

**Figure 2:**
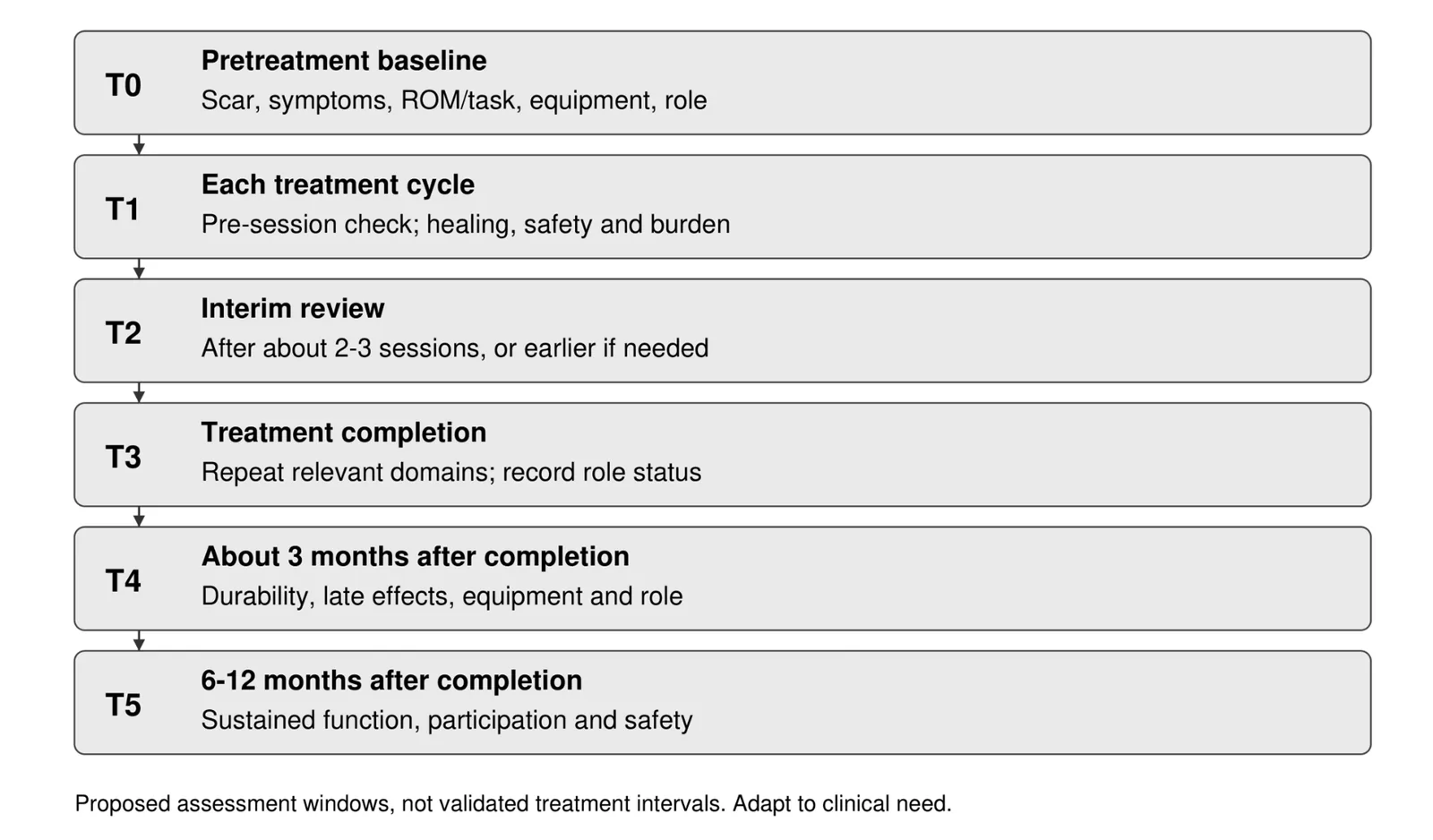
Treatment-linked follow-up pathway. T0: baseline; T1: each treatment cycle, including pre-session review and post-session healing and safety; T2: interim review; T3: treatment completion; T4: approximately three months after completion; T5: six to 12 months after completion; ROM: range of motion The image was created by Stefan Bigge and Bianca Bigge using ReportLab 4.4.9 (ReportLab, Inc., Boston, MA, USA).

Interpreting and Reporting Change

The framework does not recommend a single total score. Studies and clinical services should report baseline values, absolute change, follow-up duration, and missing data for each construct. Where a validated minimal important difference exists, the proportion meeting that threshold should be shown. When no threshold is established, responder categories should be prespecified and sensitivity analyses should test alternative definitions. Patient and observer scar ratings should be shown separately, and POSAS pain and itch should not be hidden within a total score.

For ROM, the joint, plane, active or passive method, positioning, pain, and limiting structure should be documented. A numerical gain should be linked to the predefined regional task. For equipment, the item, interface, duration, symptoms, skin findings, and task effect should be recorded. Safety should be reported per procedure as well as per patient. Role status should identify whether the person returned to the same role, returned with restrictions, changed role, or left the workforce or service, and whether the result persisted at later follow-up.

Implementation and Future Validation

A one-page form or electronic dataset could support implementation. Electronic PROM use requires attention to usability, language, accessibility, completeness, clinical workflow, and feedback [50]. Completion time for the proposed dataset has not been measured. Collection should be shared across dermatology, burn surgery, rehabilitation, therapy, pain, mental health, and occupational medicine teams.

Prospective validation should proceed in stages. First, qualitative interviews with patients, service members, clinicians, therapists, and occupational stakeholders should refine wording and establish content validity, particularly for equipment tolerance and treatment burden. Second, a multicenter feasibility study should assess completion time, missingness, interrater reliability, responsiveness, and redundancy. Third, a prospective registry should test which domains predict sustained return to role and whether CFU-level anatomy improves prediction beyond TBSA. Finally, comparative studies should evaluate laser modality, timing, repeated-session response, health economics, and clinically important change while accounting for concurrent rehabilitation and natural scar maturation.

Military and civilian systems could use a shared core dataset with context-specific role modules. A multinational retrospective study of conflict-related burns and traumatic scars could determine treatment frequency, indications, number of sessions, complications, ROM change, equipment tolerance, and duty disposition. A subsequent prospective registry could validate the framework across different health systems and occupational settings.

Limitations

This structured narrative evidence map used one bibliographic database with targeted supplementation. Anchor selection prioritized framework contribution rather than exhaustive extraction from every relevant study. The detailed selection audit was compiled during revision. Full-text review was targeted, and no separate cumulative full-text assessment count was retained. No formal study-level risk-of-bias assessment or meta-analysis was performed.

The underlying treatment literature is dominated by burn scars, AFL, PDL, single-center cohorts, and United States or other high-resource settings. Direct functional evidence remains comparatively small, and improvements may reflect concurrent therapy, natural maturation, selection, or regression to the mean. Military RTD evidence is limited, and civilian return-to-work results do not transfer automatically to military deployability or specific occupational roles.

The proposed framework was synthesized from published evidence and clinical reasoning but was not developed through patient consensus, formal Delphi methods, or psychometric validation. Equipment tolerance and the proposed graded role categories are particularly exploratory. The framework should therefore be tested and refined before being represented as a core outcome set, clinical standard, or decision rule.

## Conclusions

Laser-scar rehabilitation should be assessed through symptoms, regional function, treatment burden, safety, and participation, as well as appearance. These domains should be reported separately rather than reduced to a single scar score. The proposed minimum-plus-modules framework links established measures to treatment and follow-up while retaining equipment tolerance and graded role status as exploratory domains.

The framework is a proposal for consistent documentation, not a validated core outcome set or a treatment-effect claim. Patient input, feasibility testing, psychometric evaluation, and prospective assessment of sustained return to role are required before adoption as a standard.

## Appendices

Appendix A

Search Strategy, Anchor-Selection Criteria, and Source Register

Table 6 provides the seven PubMed search blocks. Search date: July 21, 2026. No date, language, or publication-type filters were applied. Counts refer to archived exports before PMID-based deduplication.

**Table 6:** PubMed/MEDLINE search strategy. POSAS: Patient and Observer Scar Assessment Scale; VSS: Vancouver Scar Scale; PROM: patient-reported outcome measure; RTD, return to duty; ROM: range of motion

Block/scope	Records	Search string
1. Main laser, burn/traumatic scar, and outcome search	221	(("Laser Therapy"[Mesh] OR "laser therapy"[tiab] OR laser[tiab] OR lasers[tiab] OR "fractional laser"[tiab] OR "ablative fractional laser"[tiab] OR "fractional CO2"[tiab] OR "fractional carbon dioxide"[tiab] OR "CO2 laser"[tiab] OR "carbon dioxide laser"[tiab] OR "pulsed dye laser"[tiab] OR PDL[tiab]) AND ("burn scar"[tiab] OR "burn scars"[tiab] OR "postburn scar"[tiab] OR "postburn scars"[tiab] OR "post-burn scar"[tiab] OR "post-burn scars"[tiab] OR "traumatic scar"[tiab] OR "traumatic scars"[tiab] OR "scar contracture"[tiab] OR "scar contractures"[tiab] OR "burn contracture"[tiab] OR "burn contractures"[tiab]) AND (outcome[tiab] OR outcomes[tiab] OR function[tiab] OR functional[tiab] OR rehabilitation[tiab] OR "range of motion"[tiab] OR ROM[tiab] OR pain[tiab] OR pruritus[tiab] OR itch[tiab] OR itching[tiab] OR "quality of life"[tiab] OR POSAS[tiab] OR VSS[tiab] OR "Vancouver Scar Scale"[tiab] OR "Patient and Observer Scar Assessment Scale"[tiab])) NOT (acne[tiab] OR "acne scar"[tiab] OR "acne scars"[tiab] OR burnout[tiab])
2. Focused laser-plus-burn-scar evidence search	85	(("ablative fractional laser"[tiab] OR "fractional CO2"[tiab] OR "fractional carbon dioxide"[tiab] OR "CO2 laser"[tiab] OR "carbon dioxide laser"[tiab] OR "pulsed dye laser"[tiab] OR PDL[tiab]) AND ("burn scar"[tiab] OR "burn scars"[tiab] OR "postburn scar"[tiab] OR "postburn scars"[tiab] OR "post-burn scar"[tiab] OR "post-burn scars"[tiab] OR "hypertrophic burn scar"[tiab] OR "hypertrophic burn scars"[tiab]) AND (POSAS[tiab] OR "Patient and Observer Scar Assessment Scale"[tiab] OR VSS[tiab] OR "Vancouver Scar Scale"[tiab] OR "range of motion"[tiab] OR function[tiab] OR functional[tiab] OR pain[tiab] OR pruritus[tiab] OR itch[tiab] OR itching[tiab] OR "quality of life"[tiab])) NOT (acne[tiab] OR burnout[tiab])
3. POSAS, VSS, scar scales, psychometrics, and PROMs	442	((scar*[tiab] OR burn*[tiab] OR traumatic[tiab]) AND ("scar assessment"[tiab] OR "scar assessments"[tiab] OR "scar scale"[tiab] OR "scar scales"[tiab] OR "Patient and Observer Scar Assessment Scale"[tiab] OR POSAS[tiab] OR "Vancouver Scar Scale"[tiab] OR VSS[tiab] OR "SCAR-Q"[tiab] OR "patient-reported outcome"[tiab] OR "patient-reported outcomes"[tiab] OR "patient reported outcome"[tiab] OR "patient reported outcomes"[tiab] OR PROM[tiab] OR PROMs[tiab] OR psychometric*[tiab] OR validation[tiab] OR responsiveness[tiab]))
4. ROM, goniometry, contracture, and functional motion	167	((burn*[tiab] OR scar*[tiab] OR contracture*[tiab]) AND ("range of motion"[tiab] OR ROM[tiab] OR goniometr*[tiab] OR "functional range of motion"[tiab] OR "cutaneous functional unit"[tiab] OR "cutaneous functional units"[tiab]) AND (function*[tiab] OR rehabilitation[tiab] OR outcome*[tiab] OR assessment[tiab] OR measurement[tiab]))
5. Pain, pruritus, itch, and sleep outcomes	186	((((burn*[tiab] OR traumatic[tiab]) AND scar*[tiab]) OR "burn injury"[tiab] OR "burn injuries"[tiab] OR contracture*[tiab]) AND (pain[tiab] OR pruritus[tiab] OR itch*[tiab] OR sleep[tiab] OR insomnia[tiab] OR "sleep disturbance"[tiab] OR "sleep disturbances"[tiab]))
6. Military populations, RTD, and return to work	22	((burn*[tiab] OR scar*[tiab] OR contracture*[tiab]) AND (military[tiab] OR soldier*[tiab] OR "service member"[tiab] OR "service members"[tiab] OR servicemember*[tiab] OR combat[tiab] OR veteran*[tiab]) AND ("return to duty"[tiab] OR deployab*[tiab] OR "duty status"[tiab] OR "return to work"[tiab] OR function*[tiab] OR rehabilitation[tiab]))
7. Military-journal and novelty scan	68	(("Military Medicine"[jour] OR "Military Medical Research"[jour] OR "BMJ Military Health"[jour] OR "Journal of the Royal Army Medical Corps"[jour] OR "Journal of Special Operations Medicine"[jour]) AND (burn*[tiab] OR scar*[tiab] OR laser*[tiab] OR rehabilitation[tiab] OR "return to duty"[tiab] OR deployab*[tiab] OR function*[tiab]))

Appendix B

The eligibility and citation-priority criteria are summarized in Table 7.

**Table 7:** Eligibility and anchor-selection criteria. ROM: range of motion

Criterion	Operational definition
Population and applicability	Burn and non-burn traumatic scars. Broader measurement, rehabilitation, and occupational literature was eligible when it informed a defined framework construct.
Relevant domains	Laser-associated outcomes or safety; scar assessment or psychometrics; contracture, ROM, or rehabilitation; pain, itch, sleep, psychosocial burden or quality of life; equipment interfaces; return to work or duty.
Measurement studies	Scar-scale, device, and measurement-property studies were eligible when they informed a prespecified domain, irrespective of whether they evaluated laser treatment.
Scope exclusions	Animal-only studies; acne-scar reports; unrelated laser applications; acute burn care without post-acute outcome relevance; general scar, surgical, symptom or military reports without a relevant measurement contribution.
Anchor priorities	Direct comparative or functional evidence; instrument validity or reliability; established outcome frameworks; prior serial-assessment methods; distinct military or occupational information.
Selection among relevant sources	A relevant report could remain uncited when another source adequately covered its construct or synthesis, or when it added only narrow or lower-level evidence. Protocols without results were not efficacy anchors. This was not a formal risk-of-bias exclusion rule.
Direction of findings	Null, mixed, and favorable results were eligible. Statistical significance was not a retention criterion.
Audit stages	The revision-stage record audit distinguishes advancement for further anchor appraisal, selection as a cited anchor, and targeted full-text review. Further appraisal is not synonymous with full-text eligibility assessment.

Appendix C

Table 8 summarizes the record-level revision audit. The further-appraisal stage used abstracts and available full texts and is not a count of full texts read. Three measurement-methods records were retained after targeted full-text reassessment. Their previous codes are preserved in the accompanying change log.

**Table 8:** Revision-stage anchor-selection audit.

Stage	Disposition or reason	n
Identification	Records across seven blocks	1,191
Deduplication	Duplicate occurrences removed	293
Screening	Unique records screened	898
Anchor appraisal	Not advanced	594
Anchor appraisal	Advanced for further appraisal	304
Citation selection	Further appraised but not selected as cited database anchors	269
Citation selection	Database-derived anchors retained	35
Supplementation	Supplemental anchors retained	15
Synthesis	Total cited anchors	50
Not advanced: reason	Insufficient direct relevance to prespecified framework domains at title/abstract screen	324
Not advanced: reason	Symptom literature not specific to burn/traumatic scar rehabilitation	10
Not advanced: reason	Animal/preclinical study	16
Not advanced: reason	Reconstructive/surgical technique without distinct measurement-framework contribution	84
Not advanced: reason	Non-target scar population or general scar literature without direct burn/traumatic applicability	146
Not advanced: reason	Military/operational topic unrelated to post-acute scar rehabilitation	9
Not advanced: reason	Acute burn care without post-acute scar outcome relevance	5
Not advanced: reason	Subtotal	594
Not selected as anchor: reason	Overlapping review/synthesis; more directly relevant or newer anchor selected	30
Not selected as anchor: reason	Protocol/no completed outcome data	8
Not selected as anchor: reason	Narrow symptom/safety evidence; domain represented by stronger or more directly applicable anchor	29
Not selected as anchor: reason	Treatment-focused evidence without unique framework contribution or redundant with selected anchors	106
Not selected as anchor: reason	Lower-level case report; stronger or broader evidence available	25
Not selected as anchor: reason	Relevant measurement/rehabilitation evidence but construct already represented by selected anchor	67
Not selected as anchor: reason	Contextual participation/military evidence without unique burn-scar framework contribution	4
Not selected as anchor: reason	Subtotal	269

Appendix D

Table 9 identifies each cited source and its contribution. Database-derived denotes presence in the archived seven-block PubMed exports; supplemental denotes sources outside that set identified through targeted supplementation or reference checking. The register comprises different evidence types, not 50 distinct efficacy studies.

**Table 9:** Cited anchors and their contribution.

Ref.	Source/identifier	Source group	Contribution to the framework
1	Perez KG et al. PMID: 35400523 10.1016/j.burns.2022.03.011	Supplemental	Context for military burn burden and rehabilitation needs.
2	Joint Trauma System Burn care guideline	Supplemental	Scope of deployed/acute burn guidance; separates post-healing measurement from acute doctrine.
3	Riva HR et al. PMID: 41642145 10.36849/JDD.9261	Database; block(s) 1	Direct military laser prior art.
4	Shumaker PR PMID: 25922953 10.12788/j.sder.2015.0123	Database; block(s) 1	Established rehabilitation-oriented approach to traumatic scars.
5	Anderson RR et al. PMID: 24336931 10.1001/jamadermatol.2013.7761	Database; block(s) 1;5	Traumatic scar laser consensus and prior recommendations.
6	Seago M et al. PMID: 31820478 10.1002/lsm.23201	Supplemental	International scar/contracture laser recommendations.
7	Leszczynski R et al. PMID: 36161591 10.1002/14651858.CD011642.pub2	Supplemental	Comparative evidence and published certainty assessment.
8	Buhalog B et al. PMID: 32926192 10.1007/s00403-020-02135-5	Database; block(s) 1	AFL evidence synthesis and pre-existing serial outcome recommendations.
9	Pham VX et al. PMID: 39523456 10.1002/lsm.23858	Database; block(s) 1;3;5	Extent and limitations of functional, symptom, and QoL evidence.
10	Brewin MP et al. PMID: 40319828 10.1016/j.burns.2025.107500	Database; block(s) 1;2;3	PDL comparative patient-POSAS effect and uncertainty.
11	Douglas H et al. PMID: 30846294 10.1016/j.bjps.2019.01.027	Supplemental	Reviewer-requested AFCO2L trial; symptom results and long-term limitations.
12	Lewis CJ et al. PMID: 37393760 10.1016/j.bjps.2023.06.012	Database; block(s) 1;2;3	Reviewer-requested ELIPSE trial; outcome-dependent treatment findings.
13	Heaslip V et al. PMID: 41297231 10.1016/j.burns.2025.107772	Database; block(s) 1;2	Patient experience and treatment burden in the ELABS pathway.
14	Betar N et al. PMID: 39549422 10.1016/j.burns.2024.107300	Database; block(s) 1;2;3	Serial POSAS/BBSIP, scar thickness, and safety after AFL.
15	Abouzeid C et al. PMID: 38943510 10.1093/jbcr/irae129	Database; block(s) 1;2;3;5	Null generic-outcome association; reported adjusted estimates.
16	Travis TE et al. PMID: 34291797 10.1093/jbcr/irab132	Database; block(s) 1;2	Repeated scar ratings, durometry, and active ROM during AFL.
17	Shumaker PR et al. PMID: 22847080 10.1097/TA.0b013e318260634b	Database; block(s) 1;4;5	Early direct functional and task-level observations.
18	Xi W et al. PMID: 32432374 10.1002/lsm.23262	Supplemental	Objective joint-ROM change; uncontrolled causal limitations.
19	Young A et al. PMID: 36936572 10.1136/bmjmed-2022-000183	Supplemental	Existing burn-wide outcome framework.
20	Deng H et al. PMID: 37806703 10.1016/j.pmr.2023.05.006	Supplemental	ICF-based activity, participation, and contextual domains.
21	Lin YR et al. PMID: 33918802 10.3390/ijerph18083970	Supplemental	Burn-rehabilitation core constructs.
22	Jones LL et al. PMID: 29031889 10.1016/j.burns.2017.09.004	Database; block(s) 3	Patient-priority domains including burden and sense of normality.
23	van de Warenburg MS et al. PMID: 37310702 10.1093/jbcr/irad089	Database; block(s) 3	Holistic scar assessment and shared decisions.
24	Choo AMH et al. PMID: 34250003 10.3389/fsurg.2021.643098	Database; block(s) 3;5	Scar-tool construct coverage and measurement limitations.
25	Draaijers LJ et al. PMID: 15253184 10.1097/01.PRS.0000122207.28773.56	Database; block(s) 3	POSAS development and feasibility.
26	DeJong HM et al. PMID: 27576936 10.1016/j.burns.2016.06.026	Database; block(s) 3;5	Multidimensional POSAS patient constructs.
27	Ziolkowski NI et al. PMID: 32842115 10.1097/PRS.0000000000007078	Database; block(s) 3	SCAR-Q across burn, traumatic, and surgical scars.
28	Tyack Z et al. PMID: 28902874 10.1371/journal.pone.0184452	Database; block(s) 3	BBSIP validity and responsiveness.
29	Brusselaers N et al. PMID: 20828761 10.1016/j.jss.2010.05.056	Database; block(s) 3	Clinical scar-scale coverage, patient perspective, site relocation, and prior assessment of motion and activity; historical measurement review.
30	Lee KC et al. PMID: 27574684 10.1186/s41038-016-0036-x	Database; block(s) 1;5	Objective measurement devices and combined subjective, objective, and functional assessment; heterogeneous measurement properties.
31	Klimitz FJ et al. PMID: 36300728 10.1093/jbcr/irac154	Database; block(s) 3	Standardized Cutometer acquisition and site relocation; single-rater versus average-rater reliability, without longitudinal or laser-effect validation.
32	Parry I et al. PMID: 30919903 10.1093/jbcr/irz038	Database; block(s) 4	Cutaneokinematic versus conventional goniometry.
33	Korp K et al. PMID: 25162944 10.1097/BCR.0000000000000149	Database; block(s) 4	Task-specific interpretation of functional ROM.
34	Parry IS et al. PMID: 40490904 10.1093/jbcr/iraf093	Database; block(s) 4	CFU-level anatomy and discharge ROM.
35	Parry IS et al. PMID: 40939297 10.1016/j.burns.2025.107677	Database; block(s) 4	CFU versus TBSA models; external-validation caution.
36	Oosterwijk AM et al. PMID: 27639820 10.1016/j.burns.2016.08.002	Database; block(s) 4	Contracture prevalence and measurement heterogeneity.
37	Wu A et al. PMID: 17686586 10.1016/j.burns.2007.03.015	Supplemental	QuickDASH after upper-limb burns.
38	Gittings P et al. PMID: 27606549 10.1097/BCR.0000000000000414	Supplemental	Grip and muscle-strength dynamometry.
39	Santuzzi CH et al. PMID: 38072714 10.1016/j.jphys.2023.10.010	Database; block(s) 1;5	Pain/pruritus outcomes and intervention heterogeneity.
40	Elman S et al. PMID: 19995367 10.1111/j.1365-2133.2009.09586.x	Supplemental	5-D Itch Scale.
41	Carreras JP et al. PMID: 41649169 10.1093/jbcr/irag012	Database; block(s) 3	Symptom-response heterogeneity and baseline severity.
42	Mandavalli L et al. PMID: 40448279 10.1093/jbcr/iraf103	Database; block(s) 3	Mental-health and medication context for symptom trajectories.
43	Zhang CQ et al. PMID: 32681723 10.1093/jbcr/iraa118	Database; block(s) 1;3;5	Procedure burden, sedation recovery, and workflow.
44	Zilinskas K et al. PMID: 39087666 10.1093/jbcr/irae146	Database; block(s) 1;2;5	Antibiotic exposure and postlaser infection.
45	Chapman TT et al. PMID: 18695607 10.1097/BCR.0b013e3181848b41	Database; block(s) 6	Return-to-duty after burns; injury and functional context.
46	Niedzielski LS et al. PMID: 25501771 10.1097/BCR.0000000000000148	Database; block(s) 6	BSC-SS and study-specific RTD discrimination.
47	Zalneraitis BH et al. PMID: 34061814 10.1097/CORR.0000000000001840	Supplemental	RTD is not interchangeable with full functional recovery.
48	Davis SSA et al. PMID: 42101249 10.1093/milmed/usag205	Supplemental	Heterogeneous military trauma PRO/PROM literature.
49	Mason ST et al. PMID: 22138806 10.1097/BCR.0b013e3182374439	Supplemental	Civilian return-to-work context.
50	Meirte J et al. PMID: 39600000 10.3390/ebj3020025	Database; block(s) 3	Electronic PROM use and workflow.
